# Vaccine against herpes zoster

**DOI:** 10.1590/S1679-45082013000100026

**Published:** 2013

**Authors:** Jacyr Pasternak

**Affiliations:** 1 Hospital Israelita Albert Einstein, São Paulo, SP, Brazil

**Keywords:** Vaccination, Herpes zoster, Adult

## Abstract

The herpes zoster vaccine is made using high doses of live attenuated varicella/zoster virus. The vaccine is well tolerated and has few adverse effects: the most common one is pain at the injection site. Complications can occur mainly in persons who had prior zoster keratitis or uveitis. The vaccine can prevent this disease with low mortality but high morbidity.

## RESUMO

A vacinação contra o herpes-zóster é feita com altas doses de vírus vivo da varicela/zóster atenuado. A vacina é bem tolerada e tem poucos efeitos colaterais, sendo o mais comum a dor no local de vacinação. Complicações vacinais podem ocorrer, particularmente em quem já teve queratite ou uveíte por herpes zóster. A vacina pode prevenir uma doença de baixa mortalidade mas de morbidade não desprezível.

## VACCINE AGAINST HERPES ZOSTER

Herpes zoster (HZ) is caused by the same virus that causes varicella. The virus is usually reactivated in the presence of immune changes because of a disease or immunosenescence^(1)^, which seems to be inevitable for human beings and comes with ageing. Vaccine against varicella is not enough to prevent HZ in a vaccine formulation for children^(2,3)^. However, a vaccine with the same live attenuated virus but with a high amount is already available to prevent HZ in developed countries.

The mortality rate from HZ is small, almost negligible^(4)^, and could justify arguments against use of the vaccine in Brazil, particularly for the presence of other more relevant health problems. However, HZ is responsible for reasonable risk in morbidity. Neuritis after HZ is not rare when it affects elderly people; it also compromises their quality of life because of the long-term treatment and expensive medicines that often have low efficacy^(5)^. HZ ophthalmicus may cause severe eye lesions and loss of vision^(6)^. Perhaps, the vaccine should not be available for all populations, but it could be useful in specific groups such as people older than 60 years and in patients with secondary immunodeficiency, leukemia, lymphomas, general malignant disease, and AIDS. All of these groups have an increased risk for HZ.

In addition, the vaccine would prevent secondary cases of HZ including disseminated HZ, which presents a worse prognosis and risk of transmission at inpatient wards or at places where many patients are treated.

Recent studies had evaluated that the HZ vaccine provides reasonable efficacy protection; approximately 50% less cases of HZ are reported in people who are vaccinated^(7)^. Adverse effects of the vaccine are not prohibitive usually pain at the site of the injection, which usually lasts for 1–2 days^(8)^.

Another risk associated with use of the vaccine is reactivation of herpetic keratitis by zoster, which is understandable, because the immune response induced by the vaccine may cause a local reaction with antigen persistence, which often occurs in the corneas of people who have had HZ keratitis^(9)^. The same mechanism could occur in varicella-zoster uveitis virus in which reactivation is possible^(10)^. Rare cases of HZ infection by the vaccine have been described, but all were milder than the classic HZ.

A recently published Cochrane review^(11)^ showed that vaccination benefits are larger in younger elderly people (60–69 years old). The number of people that should be vaccinated to prevent one case of HZ is roughly 50, which justifies the expenses of initiating vaccination. Interestingly, pain at the injection site, which is the most common unpleasant adverse effect, is reported more often among younger elderly people^(11)^.

Other vaccines against HZ that are not made with live attenuated virus, such as the recombinant glycoprotein vaccine^(12)^, may be available; however, these vaccines lack clinical trials in large populations. Systemic adverse effects are not rare and have included fever and myalgia. In addition, these other vaccines have shown higher antibody levels than vaccines containing the live attenuated virus. Perhaps in the future, a vaccine not containing the live attenuated virus but, instead, containing recombinant proteins, which could be easily manufactured on a large scale, would lead to a cheaper and more efficient product. Unfortunately, this development probably will not be seen for a few years because clinical trials and long-term follow-ups would need to be conducted. Important data for any HZ vaccine are duration of immunity and the necessity to inject new doses of the vaccine in the future.

Vaccination is considered by several physicians as something that would interest pediatricians and, sometimes, physicians who vaccinate teenagers with the human papillomavirus vaccine, but physicians of other specialties may be less interested. For this reason, adult populations have not been vaccinated every 10 years against tetanus and diphtheria as they should. In addition, adults with whooping cough could transmit the cough to children, including infants who have a higher mortality risk for whooping cough; therefore, the acellular pertussis vaccine should be given to all adults^(13)^.

Nowadays, immunization of those taking care of adults, particularly those caring for elderly people, is important. Particularly vital is immunization against pneumococcus and hepatitis A and B for people who were not naturally immunized by asymptomatic infections. Finally, it is critical to vaccinate people against HZ when this vaccine becomes available in Brazil.
